# Anticancer effect obtained against MCA205 sarcoma following treatment with the oncolytic peptide LTX-315 in combination with immune checkpoint inhibitors

**DOI:** 10.1186/2051-1426-3-S2-P375

**Published:** 2015-11-04

**Authors:** Öystein Rekdal, Takahiro Yamazaki, Baldur Sveinbjornsson, Andrew Saunders, Laurence Zitvogel

**Affiliations:** 1 Lytix Biopharma, Oslo, Norway; 2 Gustave Roussy Cancer Campus, Villejuif, France; 3 University of Tromsö, Tromso, Norway; 4 Lytixbiopharma, Oslo, Norway; 5 Gustave Roussy Cancer Campus, Villejuif, France

## Background

Host defense peptides (HDPs) are naturally occurring molecules found in most eukaryotic species, in which they play a significant role in the first line of defense against pathogens. Several HDPs have shown to possess anticancer activity. Structure-anti-cancer activity relationship studies on the HDP bovine lactoferricin has resulted in the design of a short oncolytic 9-mer peptide, LTX-315. Mode of action studies indicate that LTX-315 induces release of tumour antigens and potent DAMPS by distortion of intracellular organelles in cancer cells. Animal studies have demonstrated that intratumoural (i.t.) treatment of several different types of syngeneic murine tumours with LTX-315 resulted in complete tumor regression and tumor specific immune responses. In the present study, we examined the potential synergistic effects of using LTX-315 in combination with anti-PD1 or anti-CTLA4 blockade.

## Methods

C57BL/6 mice with MCA205 sarcomas were first treated i.p. with either anti-PD1 Ab or anti-CTLA4 Ab followed by intra-tumoural administration of LTX-315.

## Results

Intratumoural (i.t.) administration of LTX-315 resulted in a synergistic antitumour effect when combined with i.p. administration of anti-PD1 or anti-CTLA4 compared to the effect of either the immune checkpoint inhibitors or LTX-315 alone. A synergistic effect was also achieved against distant non-treated lesions with local administration of anti-CTLA4 Ab (s.c.) and LTX-315 (i.t.) in combination.

## Conclusions

Our results demonstrate that LTX-315 in combination with anti-CTLA4 or anti-PD1 results in a synergistic antitumour effect *in vivo*. Thus, a combination therapy with the oncolytic peptide LTX-315 that mounts specific T cell responses against released tumour antigens and immune checkpoint inhibitors that can augment the tumour specific T cell responses represent a promising combination strategy in future cancer therapy. One Phase I study with LTX-315 is completed and a 2^nd^ is ongoing. Combination studies together with immune checkpoint inhibitors are planned.

